# Commentary: Negative association between Body Roundness Index and bone mineral density: insights from NHANES

**DOI:** 10.3389/fnut.2026.1733508

**Published:** 2026-03-12

**Authors:** Minghao Guo, Shengyan Yu, Yan Xue, Suping Ma, Yun Jiang

**Affiliations:** Shandong Hospital of Traditional Chinese Medicine, Jinan, China

**Keywords:** BMD, body mass index (BMI), bone health, BRI, National Health and Nutrition Examination Survey (NHANES)

## Introduction

We read with great interest the article entitled “*Negative Association Between Body Roundness Index and Bone Mineral Density: Insights from NHANES*” by Ding et al. (1). Using nationally representative data from the National Health and Nutrition Examination Survey (NHANES), the authors applied weighted regression models and smooth curve-fitting techniques to examine the association between Body Roundness Index (BRI) and bone mineral density (BMD). However, the significant reversal in the direction of association during model adjustment raises concerns about model dependency and casts doubt on the robustness of the reported negative correlation.

## Sensitivity of the BRI-BMD association to model specification

The authors constructed three weighted regression models to examine the association between BRI and BMD. Notably, the estimated association exhibited a marked direction reversal across sequential adjustments, shifting from a non-significant relationship in the unadjusted model to a positive association after partial adjustment, and ultimately to a statistically significant negative association in the fully adjusted model. Such direction switching raises concerns regarding model dependency and statistical instability, suggesting that the reported inverse association may be contingent upon covariate structure rather than reflecting a stable independent effect of BRI.

Given that BRI is derived from waist circumference and height, whereas BMI is calculated from weight and height, substantial anthropometric overlap exists between these indices. Because BMI is well-established to correlate with BMD, simultaneous adjustment for correlated adiposity measures may introduce multicollinearity and suppression effects. Under a suppression structure, the emergence of a negative coefficient for BRI could reflect redistribution of shared variance rather than a true inverse biological association.

To further elucidate this issue, multiple targeted analyses are required. Firstly, conducting a partial correlation analysis between BRI and BMD while controlling for BMI will help establish whether BRI maintains an independent association beyond BMI. Secondly, formal multicollinearity diagnostics, such as variance inflation factor (VIF) analysis, should be employed. Thirdly, reporting standardized regression coefficients (standardized β values) would enable direct comparison of the relative contributions of BRI and BMI to BMD.

## Incremental predictive value and clinical risk stratification

Although this study confirmed a statistically significant association between BRI and BMD, statistical significance alone does not establish clinical utility. Before incorporating BRI into routine assessment, it must demonstrate incremental predictive value beyond established obesity metrics.

Accordingly, nested prediction models with identical baseline covariates should be constructed: (1) baseline model + BMI; (2) baseline model + BRI; and (3) baseline model + BMI + BRI. The added value of BRI should be quantified using changes in discrimination (ΔAUC), calibration, and reclassification metrics such as NRI and IDI. Without such evaluation, the clinical added value of BRI cannot be substantiated and may simply reflect redistribution of information already captured by conventional adiposity indices.

While conventional regression-based nested models are sufficient to assess incremental value, regularization approaches (e.g., LASSO) may help address potential multicollinearity between BRI and BMI, and tree-based methods may explore nonlinear relationships (2). Nevertheless, irrespective of modeling technique, the central question remains whether BRI meaningfully enhances predictive performance. Only when such improvement is demonstrated can integration into nurse-led screening and follow-up pathways be justified to support evidence-based osteoporosis risk stratification.

## Discussion

In summary, while this study suggests an association between BRI and bone health, the reversal of direction observed in the model raises concerns regarding model dependency and statistical robustness. Before applying these findings to clinical risk stratification, it is crucial to elucidate potential multicollinearity and suppression effects, as well as demonstrate their incremental predictive value beyond established obesity measures.
